# Faithful Approaches: Considering Islamic Tenets in ADRD Care for Muslim Women

**DOI:** 10.1002/alz.087065

**Published:** 2025-01-09

**Authors:** Bilal Irfan, Ghadeer Ankouni, Jonathan M Reader, Navid Seraji‐Bozorgzad, Bruno Giordani, Kelly M. Bakulski, Arijit K Bhaumik, Benjamin M. Hampstead, Annalise Rahman‐Filipiak

**Affiliations:** ^1^ Michigan Alzheimer’s Disease Research Center, Ann Arbor, MI USA; ^2^ University of Michigan Medical School, Ann Arbor, MI USA; ^3^ University of Michigan School of Public Health, Ann Arbor, MI USA; ^4^ Research Program on Cognition and Neuromodulation‐Based Interventions, University of Michigan, Ann Arbor, MI USA; ^5^ VA Ann Arbor Healthcare System, Ann Arbor, MI USA; ^6^ University of Michigan, Ann Arbor, MI USA; ^7^ Michigan Alzheimer’s Disease Research Center ‐ University of Michigan, Ann Arbor, MI USA

## Abstract

Alzheimer’s disease and related dementias (ADRD) and its associated care pose unique challenges, particularly within minority groups such as Muslim women. This population may face higher rates of ADRD alongside barriers to accessing culturally sensitive care. This abstract emphasizes the crucial role of understanding and integrating Islamic cultural and religious practices into ADRD care. Key aspects include respecting principles of modesty and privacy, critical for women who observe hijab or niqab. There is a great heterogeneity amongst the Muslim population, spanning diverse linguistic and ethnic backgrounds, varied levels of religious observance and influence of cultural norms, and intra‐religious jurisprudential differences. Ensuring cultural sensitivity in telehealth settings, clinician‐patient interactions, and neuropsychological evaluations is paramount. Practical measures, such as providing secluded environments for virtual consultations and using educational materials featuring women in hijabs, can significantly enhance patient comfort and understanding. In clinical settings, it’s important to acknowledge some Muslim women’s preferences for female healthcare providers and minimize clothing modifications. Understanding the role of the mahram and other close family members in providing support and essential information during clinical interactions is crucial. Accommodating cultural norms during cognitive testing and other assessments is also vital for effective care. Advanced care planning for Muslim patients must be mindful of Islamic beliefs around end‐of‐life care and organ donation. The discussion of diagnostic procedures, treatment planning, and caregiving needs to be contextualized within Islamic ethics and family dynamics. Considerations such as Halal‐compliant medications and facilitating religious practices in healthcare settings are important components of patient‐centered care. This abstract highlights the necessity for healthcare providers to adopt culturally sensitive approaches that respect the values, beliefs, and practices of Muslim women with ADRD. It advocates for ongoing research and collaboration between healthcare professionals and Muslim communities to further refine these approaches. By understanding and addressing the specific needs of Muslim women, healthcare providers can offer more effective and empathetic care, thereby improving the quality of life for this underserved population in the realm of dementia care.

